# Interplay of the RNA Exosome Complex and RNA-Binding Protein Ssd1 in Maintaining Cell Wall Stability in Yeast

**DOI:** 10.1128/spectrum.00295-21

**Published:** 2021-07-14

**Authors:** Ana Novačić, Nada Šupljika, Nikša Bekavac, Bojan Žunar, Igor Stuparević

**Affiliations:** a Laboratory of Biochemistry, Department of Chemistry and Biochemistry, Faculty of Food Technology and Biotechnology, University of Zagreb, Zagreb, Croatia; University of Mississippi

**Keywords:** RNA metabolism, yeast cell wall, RNA exosome, Rrp6, Dis3, Ssd1, RNA metabolism

## Abstract

Yeast cell wall stability is important for cell division and survival under stress conditions. The expression of cell-wall-related proteins is regulated by several pathways involving RNA-binding proteins and RNases. The multiprotein RNA exosome complex provides the 3′→5′ exoribonuclease activity that is critical for maintaining the stability and integrity of the yeast cell wall under stress conditions such as high temperatures. In this work, we show that the temperature sensitivity of RNA exosome mutants is most pronounced in the W303 genetic background due to the nonfunctional *ssd1-d* allele. This gene encodes the RNA-binding protein Ssd1, which is involved in the posttranscriptional regulation of cell-wall-related genes. Expression of the functional *SSD1-V* allele from its native genomic locus or from a centromeric plasmid suppresses the growth defects and aberrant morphology of RNA exosome mutant cells at high temperatures or upon treatment with cell wall stressors. Moreover, combined inactivation of the RNA exosome catalytic subunit Rrp6 and Ssd1 results in a synthetically sick phenotype of cell wall instability, as these proteins may function in parallel pathways (i.e., via different mRNA targets) to maintain cell wall stability.

**IMPORTANCE** Stressful conditions such as high temperatures can compromise cellular integrity and cause bursting. In microorganisms surrounded by a cell wall, such as yeast, the cell wall is the primary shield that protects cells from environmental stress. Therefore, remodeling its structure requires inputs from multiple signaling pathways and regulators. In this work, we identify the interplay of the RNA exosome complex and the RNA-binding protein Ssd1 as an important factor in the yeast cell wall stress response. These proteins operate in independent pathways to support yeast cell wall stability. This work highlights the contribution of RNA-binding proteins in the regulation of yeast cell wall structure, providing new insights into yeast physiology.

## INTRODUCTION

The yeast cell wall is an essential structure that determines the shape of the cell and shields it from environmental stress (1). It is a cross-linked network composed of β-1,3-glucan, β-1,6-glucan, chitin and mannoproteins. Cell wall stability is particularly important for cell division and survival under stress conditions, so cell wall remodeling needs to be tightly regulated by regulating the expression of cell-wall-related proteins. Expression of cell wall proteins is a complex process because these proteins must be transported to the cell periphery via the secretory pathway and simultaneously with their transport undergo various posttranslational modifications. Accordingly, their expression is often regulated at the transcriptional and posttranscriptional levels by multiple signaling pathways and RNA-binding proteins (1–5).

The RNA-binding protein Ssd1 regulates the localization and suppresses translation of mRNAs encoding cell morphogenesis proteins by directing their incorporation into P-bodies and stress granules (6, 7). Ssd1 exerts its regulatory effects primarily by binding to the 5′ and 3′ untranslated regions (UTRs) of its mRNA targets, which primarily encode hydrolytic enzymes localized in the yeast cell wall (5, 6, 8). Ssd1 itself is negatively regulated by phosphorylation through the LATS/NDR protein kinase Cbk1, which is localized to regions of cell growth and cytokinesis (i.e., the bud and bud neck), where expression of cell wall hydrolases is necessary to enable cell wall remodeling and expansion (6). The *SSD1* gene is hypomorphic in a commonly used laboratory strain, W303, which accounts for a number of phenotypic differences between W303 and other strain backgrounds (9–13).

A key regulator of RNA metabolism in eukaryotic cells is the RNA exosome, an essential multiprotein complex involved in 3′→5′ RNA degradation and processing (14). Targets of RNA exosome processing include rRNAs, snoRNAs, and snRNAs, whereas the exosome degrades aberrant tRNAs, mRNAs and noncoding RNAs termed cryptic unstable transcripts (CUTs) (14, 15). Nine RNA exosome subunits form a catalytically inactive core through whose channel single-stranded RNA substrates are threaded to the processive exoribonuclease subunit Dis3 (16, 17). The only nonessential subunit of the RNA exosome, Rrp6, binds to the top of the core and provides distributive exoribonucleolytic activity (16, 17). The roles of the core subunits and Rrp6 also include stimulation of the exoribonuclease activity of the Dis3 subunit (18). We have recently shown that the exoribonuclease activity of Dis3 and a noncatalytic function of Rrp6 are involved in maintaining cell wall stability in yeast, which is why RNA exosome mutant cells display temperature-sensitive phenotypes (19). We now show that the reason that the temperature sensitivity of RNA exosome mutant cells is most pronounced in the W303 genetic background is the presence of the nonfunctional *ssd1-d* allele. A negative genetic interaction, previously identified for *RRP6* and *SSD1* genes in a large genetic screen (20), was demonstrated to stem from a synthetic phenotype of cell wall instability, since these proteins may function in parallel pathways (i.e., via different mRNA targets) to maintain cell wall stability.

## RESULTS

**FIG 1:**
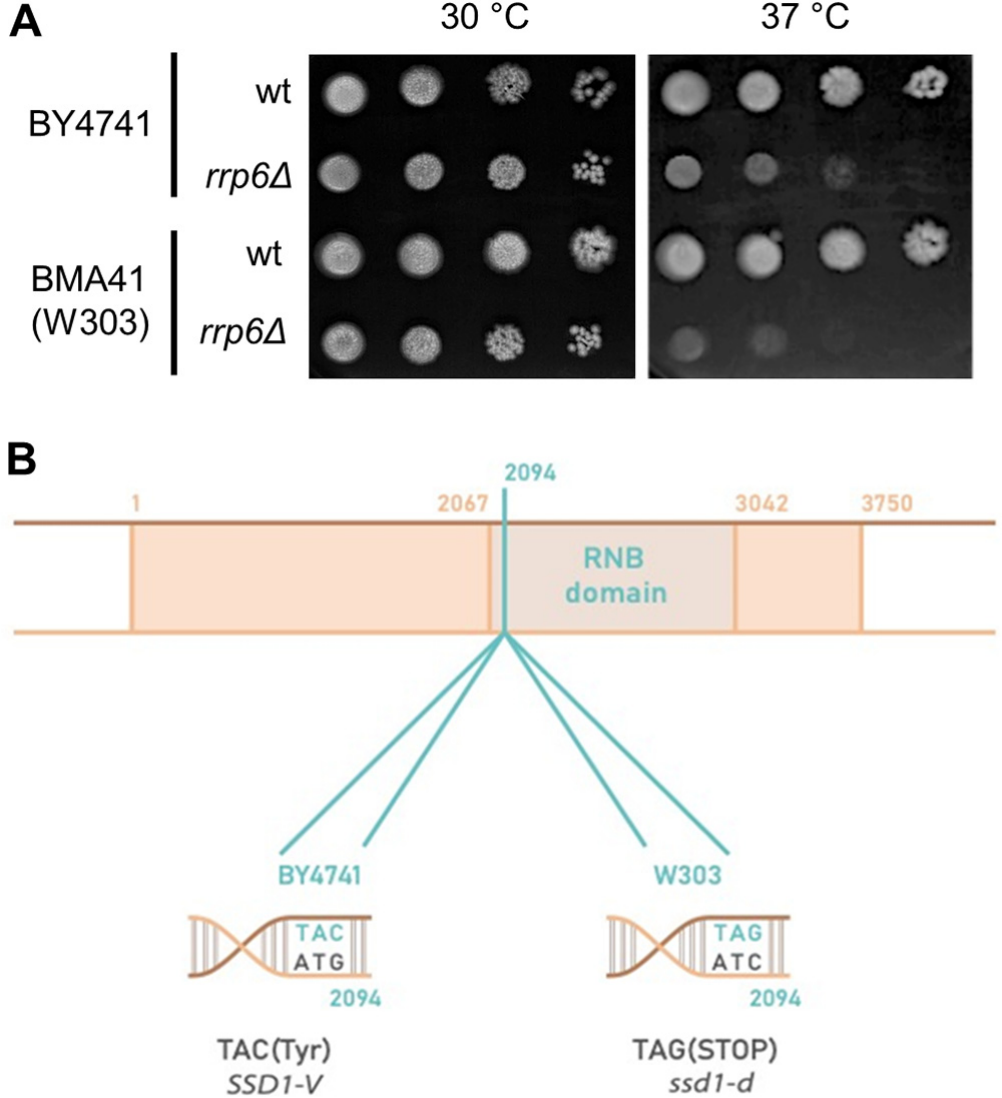
(A) The *rrp6*Δ mutation results in a stronger temperature-sensitive phenotype in BMA41 (W303) than in the BY4741 genetic background. (B) The *SSD1-V* allele of the BY4741 strain encodes a functional Ssd1 protein, whereas the *ssd1-d* allele of the W303 strain encodes a truncated nonfunctional Ssd1 protein. wt, wild type.

We recently identified the nuclear RNA exosome complex as an important yeast cell wall regulator (19). The absence of the RNA exosome catalytic subunit Rrp6 leads to cell wall instability, which manifests as cell lysis and inviability under conditions of high temperature or other forms of cell wall stress (19). The temperature sensitivity of *rrp6*Δ mutant cells can be completely suppressed at high temperatures by providing the cells with osmotic support (e.g., by adding 1 M sorbitol to the growth medium), and this suppression was found to be consistent for *rrp6*Δ mutants from different genetic backgrounds (19). However, it was striking that the temperature-sensitive phenotype of *rrp6*Δ mutant cells was most pronounced in the W303 genetic background, making it the primary choice for studying Rrp6-related phenotypes (18, 21). As exemplified in Fig. 1A, the *rrp6*Δ mutant of the BY4741 genetic background grows better at 37°C, compared with its wild-type counterpart, than is the case for the equivalent mutant of the W303-derived BMA41 genetic background. The association between the temperature-sensitive phenotype of this mutant and cell wall instability prompted us to investigate which W303-specific alleles might be enhancing this phenotype. One possible candidate was the polymorphic *SSD1* gene, which encodes the RNA-binding protein Ssd1, which binds 5′ and 3′ UTRs of mRNAs encoding cell wall morphogenesis proteins and represses their translation (5, 6). The Ssd1 family is closely related to the Dis3L2 3′→5′ exonucleases, which belong to the same RNase II/RNB family as the catalytic subunit of the RNA exosome Dis3 (22). In the genetic background BY4741, the active *SSD1-V* allele encodes the full-length protein, whereas strains of genetic background W303 carry the *ssd1-d* allele, which contains a premature stop codon due to a C-to-G transversion at nucleotide 2094, resulting in termination of the Ssd1 protein at the beginning of its RNB domain (Fig. 1B) (12). This truncation renders Ssd1 inactive: i.e., W303-derived strains behave as deficient for Ssd1 function.

**FIG 2:**
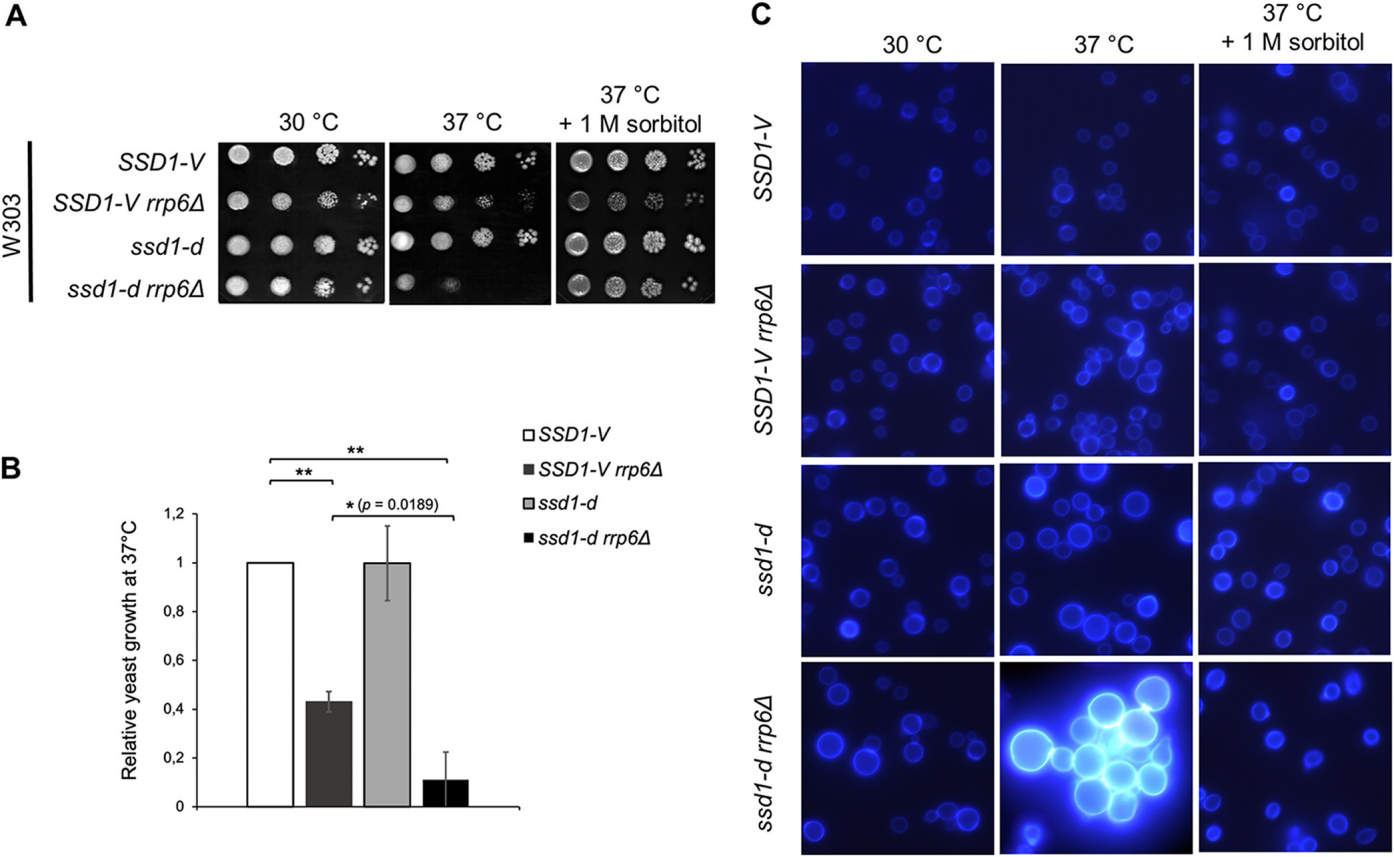
(A) The severity of the temperature-sensitive phenotype of the W303 *rrp6*Δ mutant depends on the functionality of the *SSD1* allele. (B) Strains were grown at 37°C as in panel A, and relative yeast growth on agar plates was quantified using the first dilution. The values shown represent the means and standard deviations from three independent experiments (*n* = 3). Indicated differences show the significant differences using a paired Student's *t* test. Asterisks indicate *P* values of ≤0.05 (*) and ≤0.01 (**). (C) Strains were grown as in panel A and visualized by fluorescence microscopy after calcofluor white staining of cell wall chitin.

To test whether the nonfunctional *ssd1-d* allele causes the more severe temperature-sensitive phenotype of W303 *rrp6*Δ mutant cells, we deleted the *RRP6* gene in the W303 *SSD1-V* strain and its isogenic *ssd1-d* strain and tested cell viability at 37°C (Fig. 2A). As expected, the *SSD1-V* and *ssd1-d* strains displayed normal growth at the physiological and high temperatures, whereas the *rrp6*Δ mutation impaired growth of both strains at high temperature. Crucially, the growth of the *ssd1-d rrp6*Δ mutant was significantly more impaired than that of the *SSD1-V rrp6*Δ mutant at 37°C, clearly showing that the *ssd1-d* mutation enhances its temperature sensitivity (Fig. 2B). The cell morphology of these mutants was visualized by fluorescence microscopy after calcofluor white (CFW) staining of cell wall chitin (Fig. 2C). At high temperatures, *ssd1-d rrp6*Δ mutant cells are enlarged, aberrantly shaped, grow in clumps, and stain very brightly, which indicates increased chitin deposition, whereas *SSD1-V rrp6*Δ mutant cells have a more typical shape and size under the same conditions, while still showing some defects in cell separation, as evident by the prominent staining of the cell septa. In summary, the severity of the *rrp6*Δ temperature-sensitive phenotype depends on the functionality of the accompanying *SSD1* allele.

**FIG 3:**
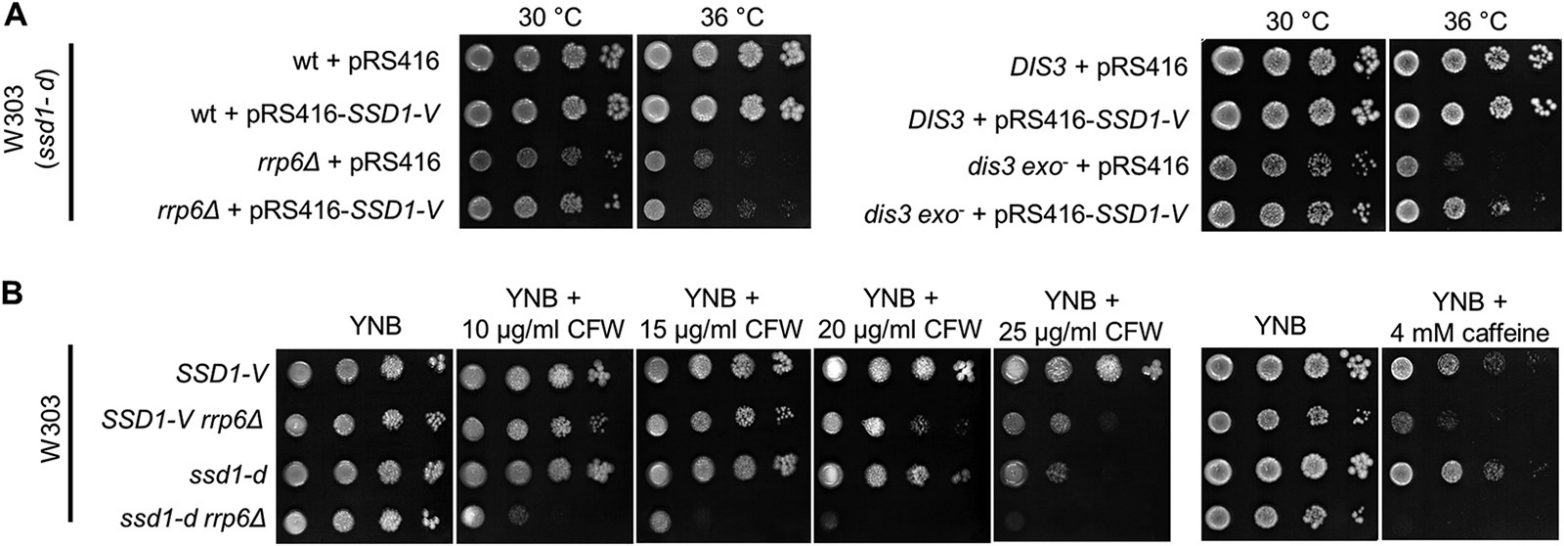
(A) Plasmid-borne expression of the *SSD1-V* allele partially suppresses the temperature-sensitive phenotype of the *rrp6*Δ mutant and the mutant for the exoribonuclease activity of the other RNA exosome catalytic subunit, Dis3. The spotting assay was performed on plates without uracil or leucine to select for the plasmid-bearing strains. (B) W303 *rrp6*Δ mutant cells are more sensitive to the cell wall stressors calcofluor white and caffeine when carrying the *ssd1-d* allele. The indicated amount of each cell wall stressor was added to plates, which were incubated at 30°C. wt, wild type.

To test whether the functional *SSD1-V* allele is also able to partially suppress the temperature-sensitive phenotypes of RNA exosome mutants when expressed from a plasmid, we introduced a centromeric plasmid carrying the *SSD1-V* allele into these cells and tested their viability at high temperatures. We found that *rrp6*Δ cells carrying a centromeric plasmid with the *SSD1-V* allele grew significantly better at 36°C than the corresponding cells carrying the empty plasmid (Fig. 3A). Importantly, expression of the *SSD1-V* allele partially suppressed the temperature-sensitive phenotype of *dis3 exo^−^* cells, which are defective for the exoribonuclease activity of the essential RNA exosome catalytic subunit Dis3 (Fig. 3A).

We next tested the viability of these strains at 30°C upon exposure to different concentrations of the cell wall stressors calcofluor white (CFW) and caffeine (Fig. 3B). Exposure to CFW or caffeine severely impaired growth of the *ssd1-d rrp6*Δ mutant, while growth of the *SSD1-V rrp6*Δ mutant was much less affected. Interestingly, *ssd1-d* and *SSD1-V rrp6*Δ mutants, in which either Ssd1 or Rrp6, respectively, is nonfunctional, were less sensitive to CFW than the *ssd1-d rrp6*Δ double mutant, in which both proteins are nonfunctional. The synthetically sick phenotype of the double mutant cells upon cell wall stress suggests that Ssd1 and Rrp6 proteins function synergistically to promote cell wall integrity.

**FIG 4:**
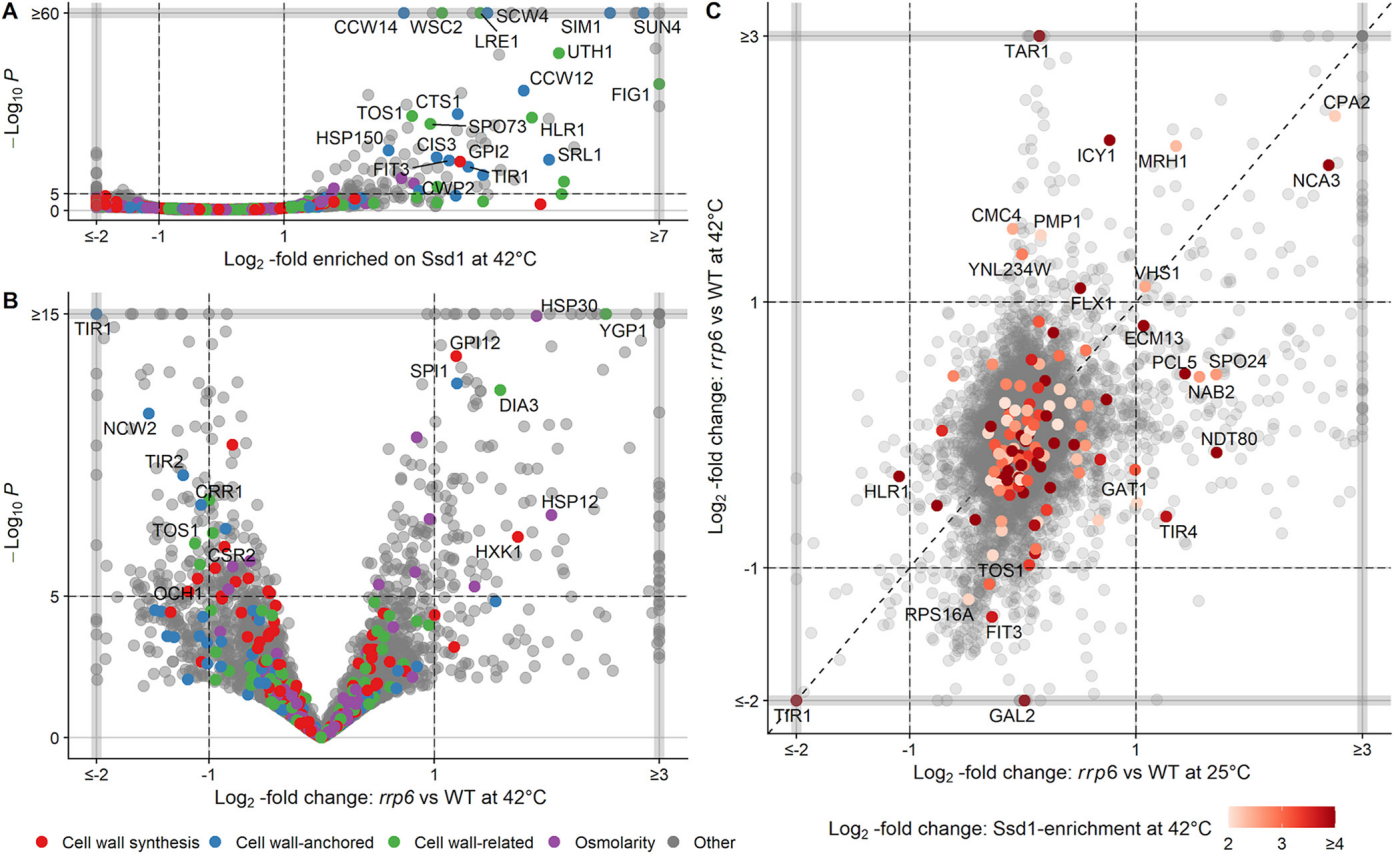
Volcano plots (A) showing which mRNAs are enriched for Ssd1 binding after heat stress (42°C for 16 min) (24) and (B) comparing transcriptomes of *rrp6*Δ versus wild-type (WT) yeast after heat stress (42°C for 10 min) (23). Different colors highlight four groups of cell-wall-associated proteins (see supplemental material). Only mRNAs belonging to these four groups of genes and more than 2-fold enriched/upregulated or depleted/downregulated (|log_2_| fold change of ≥1, −log_10_ *P* ≥ 5) are highlighted. Genes within gray lines are more up- or downregulated than shown but were placed within the lines to save space. (C) Comparison of *rrp6*Δ versus wild-type yeast transcriptomes at 25°C (*x* axis) (23) and 42°C (*y* axis, 10 min) (23), with mRNAs highlighted in the shades of red if they are enriched for Ssd1 binding more than 4-fold (log_2_ fold change of ≥2) during heat shock (42°C for 16 min) (24) and labeled only if they are up- or downregulated by more than 2-fold (|log_2_| fold change of ≥1).

Double mutant cells in which Rrp6 and Ssd1 are inactivated show a more severe cell wall instability phenotype than corresponding single mutant cells. The most straightforward interpretation of this phenomenon is that the Rrp6-containing RNA exosome and Ssd1 operate through parallel pathways—that is, by regulating different cell-wall-related transcripts. However, since Rrp6 and Ssd1 are involved in RNA decay and translational repression of mRNAs, respectively, another possibility is that they regulate a common set of cell-wall-related transcripts. Inactivation of both proteins would then have a sequential, rather than a parallel, effect on cell wall instability at high temperatures. To this end, we examined relevant transcript levels in the RNA sequencing data set of BY4741 *rrp6*Δ mutant cells (10 min at 42°C) (23) and the enrichment of Ssd1 binding to mRNAs from the CRAC (cross-linking and analysis of cDNAs) data set (16 min at 42°C) (24). The first question we asked was how many cell-wall-related transcripts are significantly enriched for Ssd1 binding at high temperature? The second was how many are significantly dysregulated in *rrp6*Δ mutant cells compared with wild-type cells under similar conditions? The pool of mRNAs we focused on was divided into four main categories (see the supplemental material): those encoding cell-wall-anchored proteins, those directly or indirectly involved in cell wall assembly, and those related to osmoregulation, as Ssd1 has recently been shown to bind mRNAs encoding key osmotic response regulators (9). A large number of mRNAs significantly enriched for binding to Ssd1 represented transcripts related to cell wall construction, of which transcripts encoding cell-wall-anchored proteins and proteins indirectly involved in cell wall assembly were the most abundant (Fig. 4A). This is consistent with previous studies showing that, although Ssd1 binds various mRNAs, its major targets encode proteins involved in cell wall morphogenesis (5, 6, 24), making it a more cell-wall-specific regulator. Conversely, of the significantly dysregulated mRNAs in *rrp6*Δ mutant cells in comparison to wild-type cells at high temperature, only a small fraction represented transcripts related to cell wall construction (Fig. 4B). This is consistent with the direct or indirect involvement of the nuclear RNA exosome participating in the regulation of a variety of mRNA transcripts, as well as other RNA biotypes (25–27).

Second, we correlated the differentially expressed genes between *rrp6*Δ mutant and wild-type cells at 25°C (*x* axis) and at 42°C (*y* axis) and projected the enrichment of Ssd1 binding at 42°C as a color scheme on top of it (Fig. 4C). This enabled us to determine whether Ssd1 and Rrp6 share any common cell-wall-related targets. It is evident that the majority of transcripts bound by Ssd1 are not dysregulated in *rrp6*Δ mutant cells at 25 or 42°C, as they are localized in the middle quadrant of the correlation diagram. Furthermore, of the Ssd1 targets that are dysregulated in *rrp6*Δ mutant cells at both temperatures, only *TOS1*, *TIR1*, and *FIT3* encode cell wall proteins. Levels of Ssd1 targets that are also direct Rrp6 targets (i.e., are degraded by Rrp6) are expected to increase upon depletion of Rrp6; however, none of the transcripts whose levels follow this trend are related to cell wall assembly. Instead, all cell-wall-related targets shared by Ssd1 and Rrp6 are downregulated in *rrp6*Δ mutant cells compared with wild-type cells and therefore represent indirect targets of Rrp6. For example, *FIT3* encodes a glycosylphosphatidylinositol (GPI)-anchored cell wall mannoprotein involved in iron metabolism, a process regulated by the nuclear RNA exosome (28), and the *TIR1* gene, which encodes a serine-rich cell wall mannoprotein, is known to be negatively regulated by noncoding antisense transcription (29), which may explain its low mRNA levels in *rrp6*Δ mutant cells compared with wild-type cells. Thus, the levels of these mRNA targets are positively regulated by Rrp6 in wild-type cells and therefore do not explain the reason for the cell wall instability phenotype of *ssd1-d rrp6*Δ mutant cells. Taken together, this analysis shows that Rrp6 and Ssd1 do not have significantly overlapping cell-wall-related targets, strengthening the overall conclusion that these proteins act in independent, parallel pathways to maintain cell wall stability in yeast.

## DISCUSSION

In this work, we investigated the interplay between the RNA-binding protein Ssd1 and the RNase subunits of the RNA exosome complex, Rrp6 and Dis3, in the maintenance of yeast cell wall stability. Ssd1 is closely related to the Dis3L2 RNase family, but its RNB domain is inactive, so it does not degrade mRNAs, but suppresses their translation by sequestering them into P-bodies (6, 7). Since proteins of the Dis3L2 family lack the PIN domain that anchors Dis3 and Dis3L to the exosome core, they do not physically interact with the RNA exosome and have been shown to act in exosome-independent pathways (30, 31). In line with this, Ssd1 and the RNA exosome appear to act in independent pathways to promote cell wall stability in yeast, as simultaneous inactivation of both factors results in a synthetically sick phenotype of cell wall instability. This finding is further supported by our analysis of the mRNA targets of Rrp6 and Ssd1, which show very little overlap for mRNAs encoding cell-wall-related proteins and none that could explain the severe cell wall instability phenotype of the *ssd1-d rrp6*Δ double mutant. The absence of Ssd1 activates the cell wall integrity (CWI) pathway, a signaling cascade that is a major regulator of the cell wall stress response in yeast (32). Because the absence of Rrp6 and the CWI kinase Mpk1 results in an additive phenotype of cell wall instability (23), Rrp6 may act in a signaling pathway that parallels Ssd1 and CWI signaling. When a cell is confronted with environmental stress, a rapid response leading to cell wall remodeling is critical for cell survival. Accordingly, cell wall damage triggers signal transduction through multiple pathways (33, 34). Because of their roles in all types of RNA-based gene regulatory mechanisms, RNA-binding proteins are emerging as major players in the cell wall stress response in yeast (2, 3, 35).

In line with this, we demonstrate that the severity of the temperature-sensitive phenotype of RNA exosome mutants is modified according to the functionality of the accompanying *SSD1* allele: i.e., it is enhanced in the W303 genetic background, where it is nonfunctional. Due to the basal level of cell wall destabilization caused by the *ssd1-d* allele, RNA exosome mutations leading to temperature sensitivity have a more pronounced phenotype in this commonly used laboratory strain. Hypomorphism of Ssd1 in the W303 background has been shown to affect numerous yeast phenotypes in addition to cell wall stability, including aneuploidy tolerance, chronological life span, and transcription by all three RNA polymerases (9, 13, 36). Besides basic research on the RNA exosome complex, yeast cells are also used when modeling clinically relevant pathogenic mutations in genes encoding subunits of the RNA exosome complex to understand how these mutations alter its function (37). This study therefore highlights the need for careful interpretation of biological effects, especially when comparing data obtained with yeast strains of different origins.

## MATERIALS AND METHODS

### Strains, media, strain construction, and plasmids.

The yeast strains used in this study are listed in Table 1. Yeast strains were grown in yeast nitrogen base (YNB) medium (described in reference 19) supplemented with the required amino acids and uracil (80 mg/liter each), with addition of 16 g/liter agar for plates. The *RRP6* gene was deleted in W303 *ssd1-d* and *SSD1-V* strains using a disruption cassette generated by PCR with primers RRP6-Kan1 and RRP6-Kan2 (38). Transformants were selected on G418 plates (0.2 mg/ml; Sigma), and gene deletion was confirmed by PCR. pRS416-*SSD1-V* is a centromeric vector that carries the *SSD1-V* allele under regulation of its native promoter, and pRS416 is its corresponding empty vector (39).

**TABLE 1:** S. cerevisiae strains used in this study

Strain	Genotype	Source
BY4741		
Wild type	*MAT***a** *his3*Δ*1 leu2*Δ*0 met15*Δ*0 ura3*Δ*0*	43
* rrp6*Δ	BY4741 with *rrp6*::*KanMX4*	19

BMA41		
Wild type	*MAT***a** *ade2-1 ura3-1 leu2-3,112 his3-11,15 trp1*Δ *can1-100*	44
* rrp6*Δ	BMA41 with *rrp6*::*KanMX4*	38
* DIS3*	*MAT***a** *ade2-1 ura3-1 leu2-3,112 his3-11,15 trp1-1 can1-100 dis3*::*KanMX4* [pBS3269*-DIS3*, *LEU2*]	45
* dis3 exo* ^−^	*MAT***a** *ade2-1 ura3-1 leu2-3,112 his3-11,15 trp1-1 can1-100 dis3*::*KanMX4* [pBS3270-*dis3D551N*, *LEU2*]	45

W303		
* SSD1-V*	*MAT***a** *ade2-1 trp1-1 leu2-3,112 his3-11,15 ura3-1 SSD1-V*	13
* SSD1-V rrp6*Δ	W303 *SSD1-V* with *rrp6*::*KanMX4*	This work
* ssd1-d*	*MAT***a** *ade2-1 trp1-1 leu2-3,112 his3-11,15 ura3-1 ssd1-d*	13
* ssd1-d rrp6*Δ	W303 *ssd1-d* with *rrp6*::*KanMX4*	This work

### Spotting assays.

Exponential-phase cultures were adjusted to an optical density at 600 nm (OD_600_) of 1, and four 10-fold serial dilutions of that sample were spotted onto YNB plates. Plates were incubated at indicated temperatures for 3 days and photographed using a UVIDOC HD6 camera (Uvitec, Cambridge, United Kingdom). Relative yeast growth on agar plates was quantified by densitometry as described in reference 40.

### Fluorescence microscopy.

Cells were stained with calcofluor white (CFW) stain (Sigma) and visualized using the Olympus BX51 fluorescence microscope under identical conditions and settings for all samples. The fluorescence from CFW was filtered with a DAPI (4′,6-diamidino-2-phenylindole) filter.

### Bioinformatic analysis and data availability.

The bioinformatic analysis was conducted in the R computing environment (R Core Team, 2020). Comparison of the *rrp6*Δ mutant with the wild type at 25 and 42°C for 10 min was based on data from reference 23 obtained from the Gene Expression Omnibus (GEO accession no. GSE140504), while the data set quantifying Ssd1-enriched transcripts (24) was obtained from Github (https://github.com/ewallace/Ssd1_CRACanalysis_2020). Both sets of data were processed as in the original publications, using DESeq2 (41), followed by shrinking log_2_ fold change with the adaptive Student's *t* prior shrinkage estimator from the “apeglm” package (42). Detailed results of the DESeq and lfcShrink functions, used to plot Fig. 4 and exported from the R programming environment as .xlsx files, are given in the supplemental material, together with lists of genes that comprise four color-coded groups in panels A and B.
